# Investigation of the vulnerability of a partly covered karst feature in Veszprém, Hungary

**DOI:** 10.1007/s11356-020-08407-x

**Published:** 2020-04-02

**Authors:** Attila J. Trájer, Lilla Mlinárik, Tamás Hammer, Rita Földényi, János Somlai, Ákos Bede-Fazekas

**Affiliations:** 1 Institute of Environmental Engineering, University of Pannonia, Egyetem utca 10, Veszprém, H-8200 Hungary; 2 Department of Limnology, University of Pannonia, Egyetem utca 10, Veszprém, H-8200 Hungary; 3 Department of Engineering Geology and Geotechnic, Faculty of Civil Engineering, Budapest University of Technology and Economics, Műegyetem rkp. 3, Budapest, H-1111 Hungary; 4 Department of Environmental Engineering and Chemical Technology, University of Pannonia, PO Box 158, Veszprém, H-8201 Hungary; 5 Department of Radiochemistry, University of Pannonia, PO Box 158, Veszprém, H-8201 Hungary; 6 Centre for Ecological Research, Institute of Ecology and Botany, Alkotmány u. 2-4, Vácrátót, H-2163 Hungary; 7 GINOP Sustainable Ecosystems Group, Centre for Ecological Research, Klebelsberg Kuno u. 3, Tihany, H-8237 Hungary

**Keywords:** Paleodoline, Karst features, Climate change, Land use, Soil degradation, Leaching, Radionuclides

## Abstract

Karst aquifers represent the most important renewable sources of drinking water. Because anthropogenic influences threaten the integrity of karst aquifers, it is important to determine the soil erosion and karst denudation rates. In order to study the complex signs of degradation processes of the karst, a paleodoline (paleo-polje) was selected near to the county seat Veszprém, Hungary. It was found that gamma radiation measurements can be a useful tool to detect the level of soil erosion since the low gamma radiation indicates the surface proximity of the carbonate bedrock. The level of gamma radiation also predicts the potential agricultural usability of a site. Both the patterns of contamination and the erosion attack zone are strongly defined by the relief. The gullies and the deepest parts of the karstic landscape are the traps of organic materials and pollutants. The amounts of ^40^K and its decay products originate from the covering sediment and negatively correlate with the soil depth. In the case of covered karsts, the measuring of the concentration of radionuclides and field gamma-ray dose measurement together can characterize the general horizontal and vertical trends of soil erosion, the potential land use, and the vegetation.

## Introduction

Karst aquifers contribute about 25% of the total drinking water supply of the world (El Hakim and Bakalowicz 2007). In the USA, carbonate-rock aquifers provide roughly 8% of the domestic water, which was equal to the withdrawal of 23.17 million cubic meter water per day in 2000 (Maupin and Barber 2005). These freshwater-bearing karstic landscapes are highly vulnerable to the change of the climatic conditions (Ma et al. 2004), land use, and anthropogenic pollution (Kačaroğlu 1999). Global climate change threatens the water resources (Delpla et al. 2009) and highly affects the carbonate aquifer systems and the global carbonate storage of different carbonate rocks (Şen 2009; Liu and Zhao 2000). For example, severe karst rocky desertification related to human activity and possibly due to the changing climatic conditions (Sun 2010) was observed in southwestern China (Wang et al. 2004). The increasing atmospheric CO_2_ exacerbates the weathering of the carbonates (Liu and Zhao 2000) due to the increasing soluble carbonic acid content of the surface and the groundwaters. The increasing frequency and length of extreme drought episodes decrease in the density of karst vegetation and can lead to rocky desertification (Liu et al. 2015). Extreme meteorological events such as heavy rainstorms may become more frequent (Meehl et al. 2000) due to global warming and the sudden overload and suffusion of the carbonate aquifers could cause increased erosion of the covering soils and the carbonate bedrocks (Şen 2009). Soil erosion threatens the agriculture and the unexpected and suddenly opening and collapsing sinkholes may cause severe damage to the infrastructure, as well as in the livestock (Parise and Gunn 2007).

The increased porosity of the karst and the loose of the covering sediments made the aquifer more vulnerable to natural and anthropogenic pollutions. The surface and subsurface degradation of karst environments also strongly exacerbate the movement of contaminants towards the water table (Parise and Pascali 2003). In the case of the soil covered karst, the infiltration process is delayed and the infiltrating water undergoes biogeochemical processes. Due to the presence of clay-humic complexes in the soil, the dissolved polluting elements exchange and the decay of the organic compounds, the respiration of the roots, and the soil fauna produce carbon dioxide. In contrast, the open karst fractures and doline systems provide the opportunity of direct, rapid infiltration of the leaching water. In this case, any contamination reaches the karst aquifer in a quick and direct way (Marshal et al. 2013). The main anthropogenic contributors to groundwater pollution in karst areas are SO_4_^2−^, NO_3_^−^, Na^+^, K^+^, and Cl^−^ (Lang et al. 2006), but metal and metalloid (e.g., arsenic) elements (Zhang et al. 2017) and biological agents (Zhang et al. 2014) could also occur depending on the nature of the polluting industrial, agricultural, and/or public sources. The disruption of wastewater treatment in karst areas could result in the rapid decline of the quality of drinking water as it happened, e.g., in Bosnia and Herzegovina due to the Yugoslav Wars in the water catchment area of Neretva River (Calo and Parise 2009). Due to atmospheric deposition, the metal pollution can reach the karst waters far away from the sources (Schettler and Romer 2006).

Karst areas occupy about 20% of the Earth’s ice-free land area (Ford and Williams 2013). In Europe, this ratio is much higher, because about 22% of the European land surface is occupied by carbonate rocks and almost 14% of the surface of the Old Continent is covered by carbonate rock outcrops (Chen et al. 2017). Most of the carbonate outcrop areas belong to the Alpine Orogeny Belt, which mainly built up Mesozoic carbonates. The Transdanubian Range in Hungary is a typical representative of the Alpine type Mesozoic platform carbonate origin landscapes of Europe. Exhumed and covered paleokarst structures—including paleodolines—are frequent in this area (Korpas 1998; Korpas et al. 1999). The term “paleodoline” (or in other words, “paleo-polje”) refers to a karstic depression that was formed in the geological past, and then, it was buried and fossilized. However, it does not mean that paleodolines could not be active in the hydrological sense in the present times as it can be seen in the case of certain paleodolines in the Alps (Veress 2016). Covered paleodolines can be revealed partly or totally by newer erosion processes and these exhuming karst structures could be the targets of renewable karstification. It is frequent that faults cut off the paleodolines, forming erosion-facilitating structures (Linder 2005).

The Balaton Highland is a karstic landscape of the Transdanubian Range in Hungary. The Transdanubian type karsts were strongly affected by tectonic movements faulted into blocks. Due to the brecciation of the dolomite and limestone bedrock, major surface features are rare and poorly developed, but some sinkholes and dolines (ponors) occur (Bárány-Kevei 2005). Karrenfields and karstic erosion valleys are typical features of the recent karst geography of the Balaton Highland. The alteration of the loess-covered and opened karst is also characteristic of the area. The town of Veszprém, the most populated city in the heart of the region, and the surrounding settlements receive drinking water from the Mesozoic karst aquifer of the Veszprém plateau. The karst aquifers of the Balaton Highland are different Triassic carbonates and can be divided into a Seisian and Karnian aquiclude (Lovász and Gyenizse 2012). Certain areas of the northeastern part of the Balaton Highland show the signs of pronounced karst desertification, e.g., the so-called Gelemér Plateau east to the county seat Veszprém City.

The degradation of the karst areas in Europe has a long history. It is known that in the Mediterranean, the decline of the highly fragile karst ecosystems started in the Greek and Roman times (Parise and Gunn 2007). The environment of the Transdanubian Range was affected by the anthropogenic influence from the Paleolithic (Mészáros and Vértes 1955; Dobosi and Vörös 1979) and it was populated and became arable land in the Roman times (Firnigl 2012). The deforestation of the karst plateau occurred in the Middle Ages and the affected area lost its loess and soil cover only in the dawn of the historical New Age. It the last few centuries, the increasing human activities have had a significant impact on the karst areas of the Transdanubian Range (Móga et al. 2017; Móga et al. 2013). The inappropriate use of the open and covered karst lands combined with the inadequate agricultural practices resulted in degraded soils and the pollution of the groundwater (Móga et al. 2017).

It is known that several ancient cities of Europe and the Middle East were dependent on the water of natural karst springs. In the Roman world, aqueducts carried the freshwater from the karst springs to the urban centers (Parise and Sammarco 2015). These remarkable hydraulic works created the basement of the foundation of large cities in such arid regions like North Africa and the Middle East and other summer-dry regions of the Mediterranean Basin (Bakalowicz et al. 2002). For example, the total length of the Aqua Augusta in the Bay of Naples was 140 km including the branches (Keenan-Jones 2010). While the Roman Emperors strictly protected the karst springs, the decline of the highly fragile karst ecosystems of the Mediterranean still started in the Greek and Roman times (Parise et al. 2009). It is likely that very similar karst degradation processes occur in the present times in the karst regions of the World which caused the demise and depopulation the ancient Ephesus (Ἔφεσος) City in Asia Minor (Delile et al. 2015). Paleodolines can preserve the fingerprints of the past geological processes which makes them suitable for studying the impact of environmental influences on the karst. While several localities of the world are characterized by a high frequency of paleodolines (e.g., Mahran and Hassan 2019), the vulnerability of these features to natural and anthropogenic effects previously has not been studied.

## Aims and scope

The subject of the study was a paleodoline which is a common karst feature in karst regions. The studied karst structure can be found in an area where the mosaic of the covered and uncovered karst characterize the landscape. The aim of the present study was to study how topography and the presence/absence status of the covering sediments influence the spatial patterns of degradation processes and the accumulation of pollutants in the karst. It was also aimed to synthesize the results of the field gamma-ray dose measurements with other environmental conditions.

## Materials and methods

### Terminology

In the present work, we used the term “paleodoline” for the nomination of the studied (partly) fossilized karst depression. The terms of “sinkhole” and “paleodoline” in the European literature mean medium-sized, generally “dry” karstic depressions while in the American literature, “sinkhole” is applied in the sense of collapsing paleodoline or cover paleodoline (Sauro 2003). Gams (2000) refers to paleodolines that “are the effects of a local accelerated solution.” Sometimes, paleodolines preserved the remnants of the former laterite soils and can contain bauxite or other bauxitic materials (Bruxelles et al. 2007; Molina et al. 1994).

### The study site

**Fig. 1:**
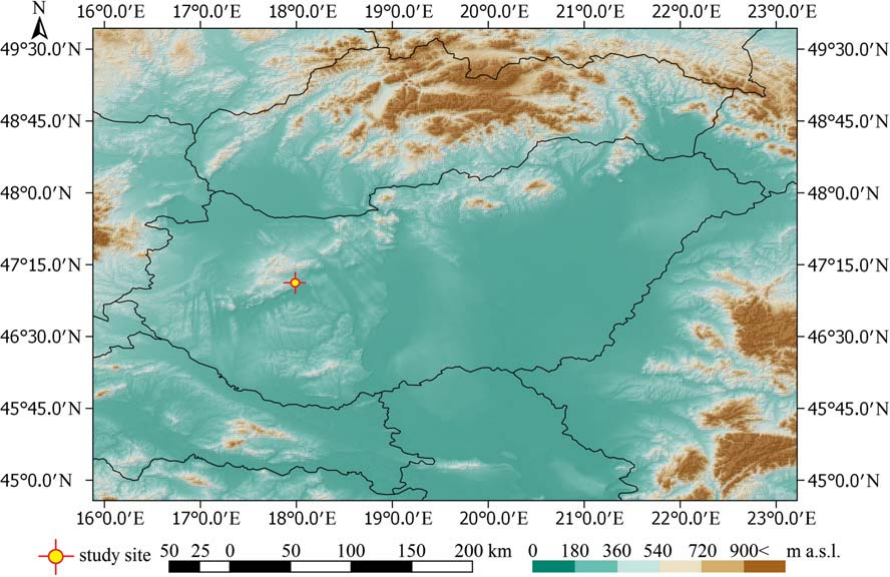
The geographical position of the Meggyespuszta paleodoline in Hungary

**Fig. 2:**
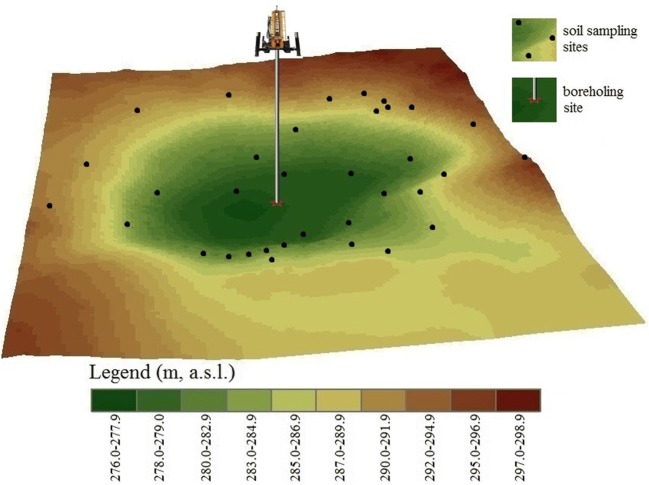
The topographic reconstruction of the Meggyespuszta paleodoline from the south view and the illustration of the site of the borehole pit and the soil sampling sites

The Meggyespuszta paleodoline is a partly fossilized karstic depression of the Transdanubian Central Range (Trájer et al. 2015); Fig. 1). In this area, the major phases of karstification occurred in the Early Cretaceous, the Latest Tertiary (Neogene), and the Pleistocene periods (Jakucs 1977). However, the Meggyespuszta paleodoline plausibly was formed during the Miocene epoch; there are some signs of a more recent cryptokarstification activity (Trájer et al. 2015). The coordinates of the center of Meggyespuszta paleodoline are N47.06111 and E17.93611 in decimal. The diameter of the object is about 820 m from the north to south and 709 m in the east-west direction. The mean diagonal diameter is about 800 m, and the mean radius is about 400 m. The average depth of the paleodoline from the visible rim is − 21 m under the narrow environment. A flat erosional valley connects to the paleodoline from the south with a southeast axis (Fig. 2).

**Fig. 3:**
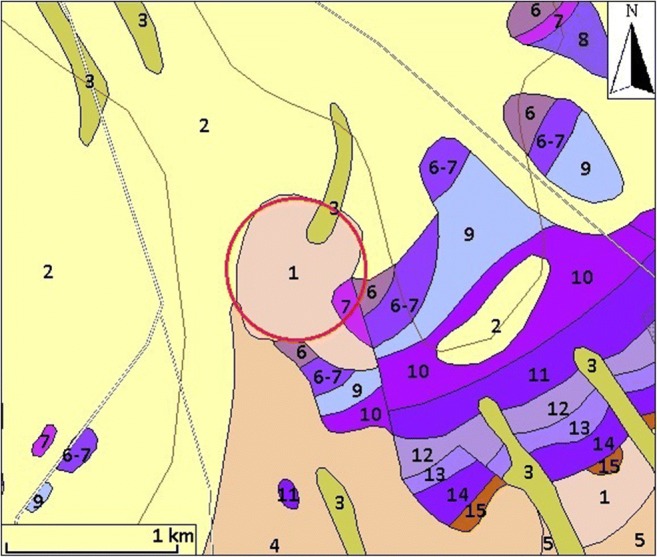
The overview map of the surrounding surface geological formations of the Meggyespuszta paleodoline (red circle) according to the surface geological map of Gyalog and Síkhegyi (2005). 1: 100000, 1–5: Holocene-Pleistocene sediments, 6–14: Triassic carbonates, and 15: upper Permian sediments. This geological map does not indicate the semi-consolidated carbonate debris at the south rim

Megyespuszta paleodoline is a mainly buried and fossilized karst structure. The carbonate bedrocks are about 230–200 million years old dolomite and limestone rocks. While Pleistocene loess covers the northern and western rims, Middle Triassic carbonate outcrops bound the east and the southwest part of the paleodoline. Figure 3 shows the overview surface geological map of the environment of the paleodoline. In the southern part, dolomite/limestone debris can be found which probably is the witness of an earlier erosion phase, when the relief was higher and the climate more humid and warmer than in the present era. The geomorphological evolution of the paleodoline had previously been studied and three main phases of the landscape evolution were described (Trájer et al. 2015). While in the surrounding area, the erosion cleared previously deposited Neogene and partly the Quaternary sediments, we concluded that the paleodoline as a “time capsule” preserved the major environmental changes of the past in the studied area (Trájer et al. 2015) (Table 1).

**Table 1:** The central coordinates of the scored 1 km^2^ areas and the calculated urbanization intensity (UI) values

Sites	Lat	Lng	Control	UI
1	47.070	17.923	–	− 0.943
2	47.070	17.937	–	0.991
3	47.070	17.950	–	− 0.428
4	47.061	17.923	–	− 0.481
5	47.061	17.937	–	− 1.182
6	47.061	17.950	–	− 0.251
7	47.051	17.923	–	− 0.466
8	47.051	17.937	–	− 1.154
9	47.051	17.950	–	− 1.182
Veszprém, downtown	47.094	17.910	+	− 1.182
Old forest	47.013	17.889	+	6.279

### The logical frame and background of the study

Since the complex investigation method of the vulnerability of paleodolines against anthropogenic effects (e.g., human-indicated soil degradation, agricultural pollution) preciously was not presented in the literature, it was an important task to build a logical framework that later could be used by other investigators for similar purposes. The main goals that should follow such an investigative logic are (1) the conditions of the narrower area and the karst aquifer (incl. the potential sources of pollutants) should be determined; (2) the local degradation features and pollution patterns should be determined both in horizontal and vertical sense and (3) the correlations between the geomorphologic, erosion features and the chemical and radiometric results should be synthesized in order to create general recommendations for the prevention of this kind of karst features.

The investigation of the vulnerability of the selected karst feature was conducted according to the following steps:

1. determination of the urbanization level and land use patterns of the surrounding area,
2. the determination of the geomorphological position of the karst feature according to the related karst aquifer,
3. the determination of the occurrence local soil degradation sites,
4. sampling the sediment at the bottom of the paleodoline in order to investigate the vertical patterns of leaching,
5. sampling the surface soil cover of the studied karst depression to show the horizontal distribution of the anthropogenic pollutants and
6. measuring the gamma radiation of the studied structure to study how geomorphological features correlate with the background radiation at soil level related to the covered/open status of a point in the karstic depression.
7. the synthesis of the gamma radiation patterns with the soil thickness, the potential vegetation, and the land use possibilities.

### Urbanization intensity measurement

Quantifying the degree of the urbanization intensity of the narrow environment of the paleodoline the “Urbanization Score” freeware (Seress et al. 2014; Czúni et al. 2012) was applied, which uses the available, public satellite images of GoogleMaps. The classification is based on the scoring approach introduced by Liker et al. (2008). This application downloads an image of 1 km^2^ area around a selected location, then divides it into 100 × 100 m cells and estimates the cover of vegetation, buildings, and paved road surfaces in each cell. From these cell scores, it generates five summary land-cover statistics then generates an overall “urbanization score” for the study site using principal component analysis (PCA) which function is part of the software (see Seress et al. 2014. for more details). These “urbanization scores” are suitable for objectively express an area’s level of habitat urbanization, thereby ranking study sites along an urbanization gradient. The urbanization level was calculated in 9 neighboring 1 km^2^ areas (sites 1 to 9), around the paleodoline (Table 2). The center of the paleodoline was used as the center of the whole studied area. The studied area 5 represents the major part of the paleodoline. To create a contrast for the PCA calculating process, a natural forest of the close Balatonfüred Town and the central downtown of the adjacent Veszprém City were used as controls. It is important to note that the program handles the arable lands as “natural” (non-urbanized) areas, similar to the natural forests, the pastures or the coast of the lakes.

**Table 2:** Detailed technical basement of the measurements

Detector	
Type	ORTECGMX 40–76 type HPGe
Measuring time	60,000 s
Efficiency	40%
Energy resolution	1.95 keV at 1332.5 keV
Spectrum and data recorder	
Type	Tennelec PCA-MR 8196 MCA
The measurement of the radionuclides	
40K	By 1461 keV of 40 K
226Ra	By 295 keV of 214Pb and 609 keV of 214Bi
232Th	911 ke V of 228Ac and 2614 keV of 208Tl

### The borehole and the soil sampling

A new hole was bored with a 7.5-cm diameter borehole bit about 86 m from the geometric center and 285 m from the nearest northern part of the rim (23.18% of the radius from the center). The D2013 borehole was executed on 13 December 2013. The hole reached the bedrock at 6.7 m and due to the hardness of the bedrock drilling was stopped at this depth. The core samples were described by the authors. Twenty-three samples were collected from 1 to 6.7 m in depth (to the firm rock floor). Six grams of samples was placed into airtight plastic sample holders. The 34 soil samples were collected consistently from – 50- to – 55-cm depth under the surface of the soil. The soil samples were collected into the same type of airtight plastic sample holders. The borehole intervals of the analyzed samples were approximately 50 cm. Because the samples were analyzed to understand general trends and not the history of sedimentation, it was concluded that a sampling frequency of 50 cm could be enough to satisfy this purpose.

### The karst water table

The geomorphological patterns of the karst table were determined by two sources: (1) the position and elevation of the natural karst springs and (2) the mean water level of the karst aquifer monitoring wells were used in order to create the mean water table profile of the karst aquifer. For example, the No. 229/000735 Veszprém-Meggyespuszta monitoring well itself is 291.55 m above sea level; its depth is 188 m and positioned in the southwestern edge of the paleodoline. For example, in 2006, the mean groundwater level was − 43.69 under the well (247.86 m ± 1.5 m).

### Chemical characterization of the soil and the borehole samples

The chemical composition of the samples was analyzed according to the Hungarian standards. The samples were prepared according to the MSZ 18094/9-79 Hungarian standard (soil corrosion tests—preparation of the soil and groundwater). The samples were homogenized, and the diameters of the sample pieces were less than 5 mm. The stone and gravel pieces and animal and vegetal residues were separated from the material and the purified soil sample was dried on 25–35 °C in air. The next steps were the milling and sieving (*d* = 2 mm) of the dried soil. Then the samples were stored in airtight boxes. The pH value was measured by digital pH meter (type: Testo 206-pH1-versatile pH/°C meter) from soil suspension. The measurement was performed according to MSZ-18094/10-79 (soil corrosion tests—determination of the soil and groundwater pH value). Each soil sample was measured three times, and the arithmetic mean was used to define the correct pH value. The sulfate content was measured by gravimetric analysis from the filtrate. The organic compound (organic carbon content) was analyzed by the calorimetric method according to MSZ 14043/9-82 standard (soil mechanical tests—determination of the organic matter content). The organic matter is the relation of the mass of the organic compounds and the mass of the dried soil sample (the measuring unit is %). The first step of the colorimetric method was the portion of the samples (1.0 g from each sample). Then 10 cm^3^ potassium dichromate and 20 cm^3^ cc. nitric acid solutions were carefully added to the portioned samples. Next, 100 cm^3^ distilled water was added to each sample and the solutions were cooled and filtered. At the same time, reference solutions were made from carbon standard solutions (2.5 g glucose was dissolved in 100 cm^3^ distilled water, and a color scale was made with different amounts of glucose content). Finally, the reference solutions and the solutions of the samples were compared to each other by color. The carbonate and dolomite content was measured by thermogravimetric analysis (TGA) with Derivatograph Q 1500 D type apparatus. On the recordings of the derivatograph, the changes in the mass of the samples, which occur due to the heat, were registered. The first part of the appearing double-peak (above 700 °C) belongs to the magnesium-carbonate, and the other peak means the calcium-carbonate. According to the quantitative analysis of the recordings, the dolomite and carbonate content could be determined by software.

### Measurements of radionuclides concentrations

Loessy brown forest soil and sandy loess were found to − 2.4 m under the soil level. With a relatively sharp transition, homogenous, sandy dolomite flour, and debris were found down to the bedrock formation, which was Budaörs Dolomite Formation from − 6.7-m depth. The measurements of the ^226^Ra, ^232^Th, ^214^Pb, ^214^Bi, ^228^Ac, ^208^Tl, and ^40^K content of the core samples were performed by semiconductor HPGe detector. The ^232^Th and ^226^Ra radionuclide contents were determined via their progenies: the ^226^Ra and content of the sample were calculated by the ^214^Pb, ^214^Bi, the ^232^Th from the ^228^Ac, and ^208^Tl contents. For the detailed technical basement of the measurements, see Table 1.

### Gamma-ray dose measurement at ground level

We used the gamma-ray dose measurement to map the karstic relief. Gamma-ray measurement is a useful completive tool of field geology and can be used in case the mapping several structures as subsurface karst structures (Gautam et al. 2000) or even the exploration of terrestrial impact craters (Vasconcelos et al. 2012) and impact paleodolines (Bose et al. 2013). The measuring of the gamma-ray dose of the surface soil is a useful tool in cases when the mapping of the karst and the covering or the sediment filling is requisite (Ali et al. 2011). The radioactive anomaly is the consequence of the higher radioactive isotope content of the filling or covering material. We used the Automess 6150 ADB ambient gamma-ray dosemeters (energy independent 23 keV-7 Mev. lower level 5 nSv/h) of the German Automers GmbH. We recorded 77 measuring points and ten measurements were performed per measuring points which means that the three-dimensional gamma-ray dose map was based on 770 measurements. Parallel to the field gamma-ray dose measurements, the depth of the soil, the vegetation type, and the local land use categories also were recorded.

### The analysis of the correlation of the field gamma-ray dose measurements and the soil samples

Soil samples were collected at 50–55 cm under the surface into well-sealed plastic bags on the same occasion in January of 2013. Correlation of gamma-ray dose and two edaphic parameters, the mass ratio of soil dolomite content and pH were calculated to examine whether one can infer soil development from gamma measurement. Since the sampling design of radiometric and edaphic measurements was different, interpolation of all the three studied parameters was done. The method we used was Inverse Distance Weighted of Spatial Analyst extension of ESRI ArcGIS 10.0 geoinformation software. Second power interpolation was done with 12 searching points and an output cell size of 2*10-5 decimal degrees. Also, the sampling domain of radiometric and edaphic measurements was different; therefore, the correlations were calculated only within the intersection of the convex hulls. Consequently, 205.542 points were made for the calculation of Pearson’s correlation coefficient and the significance of the correlation, which were done by the Hmisc package (Harrell and Dupont 2012) of R statistical software (R Core Team 2013).

## Results

### The urbanization intensity

**Fig. 4:**
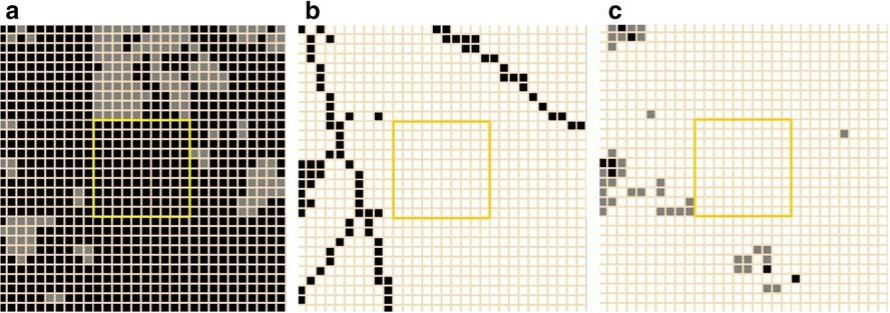
**a** Vegetation, **b** the paved roads, and **c** the buildings in the 9 km^2^ area of the Meggyespuszta paleodoline (3 × 3 km squares). Yellow squares mark the borders of the paleodoline (5.) grid. Black: > 50%, gray: < 50% cover of the area and white: the absence of the road/building structure in an area

The urbanization intensity (UI) of the squares is the following: (1) − 0.943337, (2) + 0.990694, (3) − 0.428087, (4) − 0.480731, (5) − 1.18205*, (6) − 0.251059, (7) − 0.466296, (8) − 1.15393, (9) − 1.18205. The UI squares of the control areas are the following: forest, − 1.18205; downtown of Veszprém, + 6.27889. The gained building cover also shows the eastern part of a close suburban area of Szabadságpuszta (part of Veszprém City) and the position of the close farm south to the paleodoline (Fig. 4). Asterisk marks the UI value of the paleodoline.

#### Karst water table

**Fig. 5:**
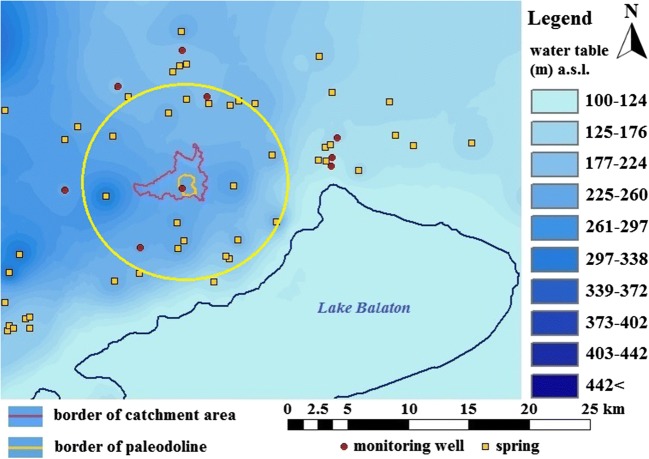
The karst and groundwater level of the east part of the Balaton Highland, the position of the springs, the monitoring wells, the rim, and the water catchment area of the Meggyespuszta paleodoline. The yellow circle indicates the possible radius of the karst system related to the paleodoline

The Meggyespuszta paleodoline can be found in the northeast part of the related karst aquifer in the Balaton Highland. According to the interpolated groundwater level map and the average groundwater level of the Meggyespuszta monitoring well, the vadose zone can be found about 26-m depth under the level of the rim of the paleodoline. The nearest springs are 5.8 km, 6.1 km, 5.6 km, 3.85 km, and 3.2 km from the paleodoline. Karst springs surround the Meggyespuszta paleodoline which seems to be the geometric center of the infiltration area of the neighboring aquifer system. The paleodiolina has an extended, asymmetrical water catchment area. Due to its hydrological, geographical position, including the characteristic circular feature of the creeks around the paleodoline, the Meggyespuszta paleodoline can be the center of an ancient local karst system with a diameter of about 16–17 km (Fig. 5).

### Chemical characterization of the borehole samples

**Fig. 6:**
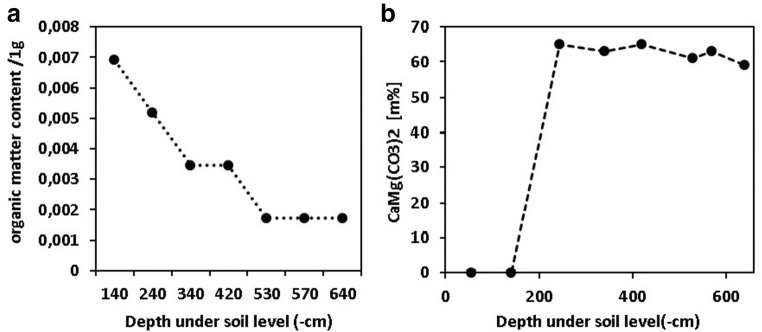
The soil organic carbon **a** and the dolomite contents **b** of the borehole samples

We found a significant negative correlation between the soil organic carbon content and soil depth (*r*^2^ = 0.93, *p* = 0.0004; Fig. 6a). We did not find significant correlation between soil depth and sulfate content (*r*^2^ = 0.18, *p* = 0.3418), pH (*r*^2^ = 0.01, *p* = 0.7906), and saline content (*r*^2^ = 0.14, *p* = 0.3997). The dolomite content of the flour samples was 64 w/w%. The brown forest soil and the loess did not contain dolomite. The dolomite content of the silt was 63 w/w%. The low standard deviation (SD = 2.34) shows the homogeneity of the material (Fig. 6b).

### The activity concentration of the borehole samples

**Fig. 7:**
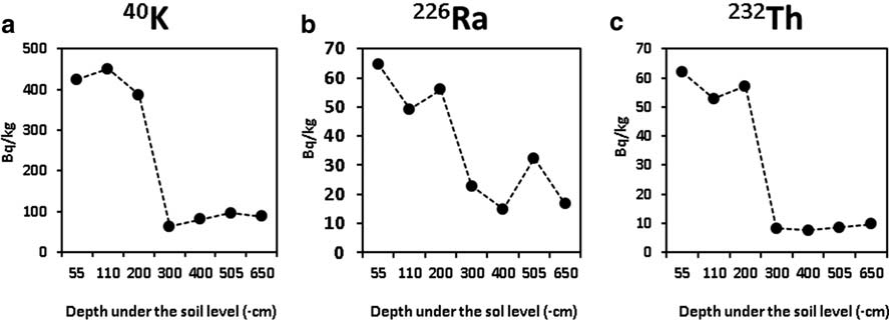
^40^K isotope activity concentration of the borehole samples **a**; the ^226^Ra isotope activity concentration of the borehole samples **b**; the ^232^Th isotope activity concentration of the borehole samples **c**

Each of the values of the measured radionuclides showed a significant decreasing trend according to soil depth in centimeters. Significant correlations were found also between soil depth and ^214^Pb content (*r*^2^ = 0.6989, *p* = 0.0190), 214Bi content (*r*^2^ = 0.6832, *p* = 0.0219), ^226^Ra content (*r*^2^ = 0.699, *p* = 0.01904), ^228^Ac content (*r*^2^ = 0.6789, *p* = 0.0227), ^208^Tl content (*r*^2^ = 0.7419, *p* = 0.0127), ^232^Th content (*r*^2^ = 0.7186, *p* = 0.0160), and ^40^K content (*r*^2^ = 0.6983, *p* = 0.0192). The measured values do not indicate the accumulation of radioactive isotopes. The 40 K isotope content of the loess and the brown forest soil (B level) and the native loess samples was five times higher (mean, 420.6 Bq/kg; SD, 32.2 Bq/kg) than of the content the dolomite flour samples of – 300- to – 650-cm depth (mean, 82.1 Bq/kg; SD, 14.1 Bq/kg). The ^40^K content rapidly decreases with a 323.0 Bq/kg /100 cm trend between − 200 and − 300 cm depth under the surface which is in accordance with the loess/dolomite silt transition level (Fig. 7a). The ^226^Ra content shows a relatively continuous decreasing trend from the surface to the local floor of the paleodoline. The ^226^Ra content of the upper three samples is 2.6 times higher than the lower four samples. We found a significant association (*r*^2^ = 0.67, *p* = 0.0237) between ^226^Ra content and the carbon content of the samples. The ^40^K content rapidly decreases with a 48.8 Bq/kg/100 cm trend between − 200 and − 300 cm under the soil level. Also, a significant correlation was found between the ^226^Ra content and the carbon content of the soil samples (*r*^2^ = 0.68, *p* = 0.0217; Fig. 7b). The ^232^Th isotope content of the loess and brown forest soil (B level) and the native loess samples are 6.65 times higher (mean, 57.2 Bq/kg; SD, 4.5 Bq/kg) than the content of the dolomite flour samples from – 300- to – 650-cm depth under soil level (mean, 8.0 Bq/kg; SD, 0.9 Bq/kg; Fig. 7c).

### The pattern of field sample’s chemistry

**Fig. 8:**
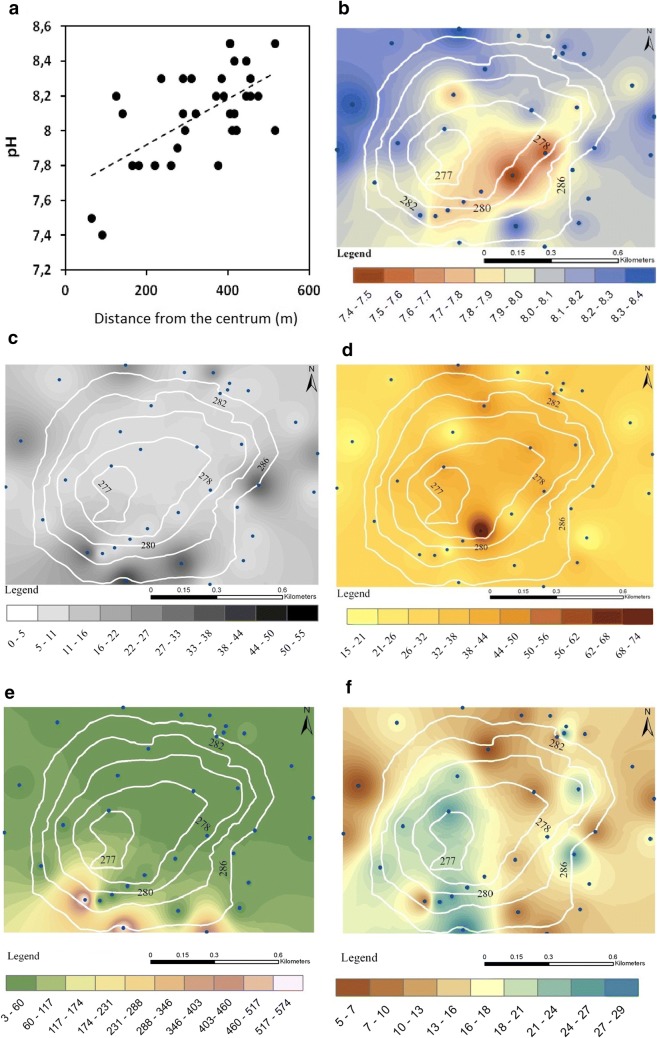
The regression of the chemistry according to the distance from the center of the doline and **a** the mapped pH values of the soil samples; **b** the mapped dolomite and **c** sulfate content of the samples **d** the mapped salinity and **e** the organic carbon content of the soil samples **f**

The chemical properties (pH) of the soil samples at 0.5-m depth show a significant positive correlation (*r*^2^ = 0.37, *p* < 0.001; Fig. 8a) with the distance from the center of the paleodoline. The mean of the pH values is mildly alkaline (pH, 7.97; SD, 0.3); the lowest pH (7.4–7.5) values were measured in the southeastern part of the paleodoline, in the remnant of the original deciduous forest. The highest pH values were measured in the margin, loess-covered areas (pH, 8.5–8.3; Fig. 8b). The dolomite content pattern is related to the carbonate outcrops in the southeastern and the southwestern part of the rim of the paleodoline and the carbonate breccia/debris in the mouth of the southern valley. The minor dolomite enrichments may refer to the presence of the exhumed carbonate bedrock (Fig. 8c). The sulfate content of the soil was relatively homogenous (mean, 0.25 w/w%; SD, 0.1 w/w%); we found only in the southern part of the paleodoline a minor enrichment (0.6 w/w%; Fig. 8d). The mean of the total soluble saline content is 0.63 w/w% (SD, 0.25 w/w%). Local enrichment of the total soluble saline content (maximum, 0.98 w/w%) was observed in the southern part of the paleodoline and the southern erosion valley (Fig. 8e). The pattern of the organic carbon concentration (mean, 1.82 w/w%; SD, 0.69 w/w%) showed a similar pattern; however, the enrichment affected rather the western and southwestern part of the paleodoline (maximum, 3 w/w%; Fig. 8f).

### Soil erosion patterns and the field gamma-ray dose results

**Fig. 9:**
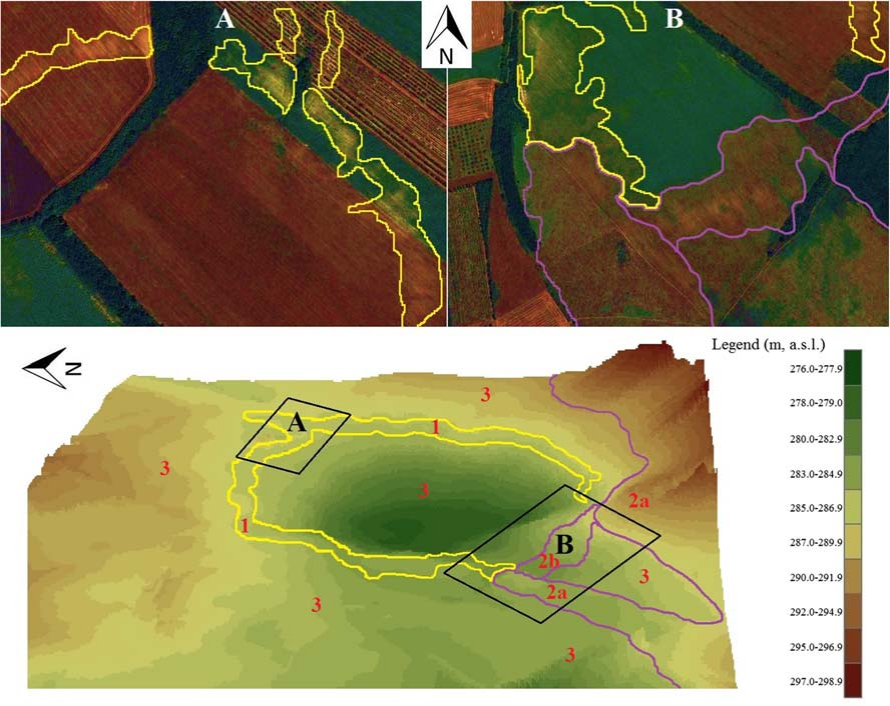
The contour lines sketch of the visible soil degradation areas from space in the oversaturated false-colored images of the northeast **a** and the south **b** part of the Meggyespuszta paleodoline. Yellow line: loess (1), purple line: dolomite or limestone bedrocks (2a) or semi-consolidated carbonate debris (2b), brow soil (3)

The degradation of the soil layer along the total length of the rim of the paleodoline is well visible already in satellite images. Along the northern two-thirds of the rim, erosion has not yet reached the bedrock. In the field visit, it was observed that both the A and the B part of the original brown soil layer disappeared from the loess parent material due to plowing and leaching. In contrast, in the southern part of the paleodoline, the dolomite/limestone bedrock and carbonate breccia has been exposed and at the west and east edges of the south palaeovalley, where only a thin layer of skeletal soil covers the surface. The south paleovalley is filled with a semi-consolidated carbonate breccia and this material appears in the surface at the southern rim of the paleodoline (Fig. 9).

**Fig. 10:**
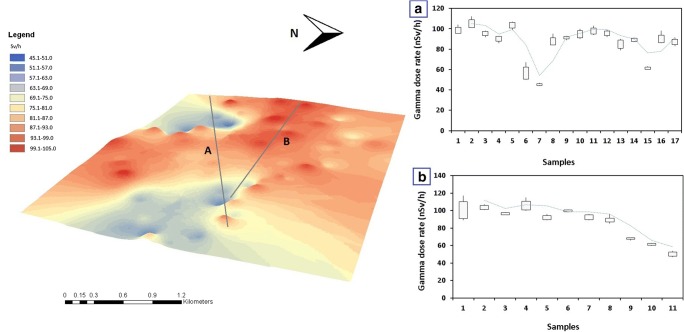
Left: The isorad map of the Meggyespuszta paleodoline based on the interpolated gamma dose rate values (nSv/h) viewed from the northeast direction; right: **a** the change of gamma dose rate (nSv/h) on from the northeast part to the east part of the rim with the moving average (blue dotted line) and the SD of the samples; **b** the change of gamma dose rate (nSv/h) on west-east direction with the moving average (blue dotted line) with the SD of the samples

We found that the gamma-ray dose at the ground level of the unburied rocks of Buchenstein Limestone Formation is about 50 nSv/h (Site 13). In the southeastern edge of the paleodoline—where the Buchenstein Formation is covered by thin soil layer (Site 11, 12)—the average gamma-ray dose rate was 61 nSv/h. The Budaörs Dolomite Formation’s gamma-ray dose is about 45 nSv/h (Site 22/A). In the southwestern rim of the paleodoline where the Budaörs Dolomite Formation is covered by thin soil layer (Site 20, 22, 36), the average gamma-ray dose was 55.5 nSv/h. The gamma-ray dose of the native loess and the loess outcroppings showed 95nSv/h on average. The gamma-ray dose of the inner areas of the paleodoline showed 93 nSv/h. Pearson’s correlation coefficient of the mass ratio of soil dolomite content and the gamma-ray dose is found to be − 0.42 with significance level *p* < 0.001 and of pH and the gamma-ray dose is found to be 0.08 with significance level *p* < 0.001. The separation of the high gamma-ray dose covered and the low gamma-ray dose opened karst areas still visible in the isorad map of the paleodoline based on the interpolated gamma-ray dose values (Fig. 10 right).

The rim of the paleodoline did not have a higher gamma-ray dose than the inner parts or the control surrounding areas. The average gamma-ray dose of the Pleistocene Loess Formation and the brown forest soil formed on the loess has about 2.11 and 1.9 times higher gamma-ray dose than the Budaörs Dolomite Formation and Buchenstein Limestone Formation itself. Outside the field, the lowest gamma-ray dose specially belongs to the rock occurrences. The south erosional karst valley of the paleodoline has statistically the same gamma-ray dose that of the deepest part of the paleodoline (Site 15, 16, 17, 18, 19) 92.7 nSv/h in average. The gamma-ray dose showed a bimodal feature on from the northeast part to the east part of the rim of the paleodoline which is related to the sites of the native rock outcrops (Fig. 10a). The narrow northern area of the paleodoline partly preserved its loess cover and shows the initial phase of soil degradation which still occurred two hundred years ago in the eastern neighborhood of Veszprém. The gamma-ray dose showed a decrease of gamma-ray dose from the western to the eastern rim of the paleodoline. The northeastern part of the paleodoline is covered by loess; the southern part of the bedrock reaches the surface (Fig. 10b).

**Fig. 11:**
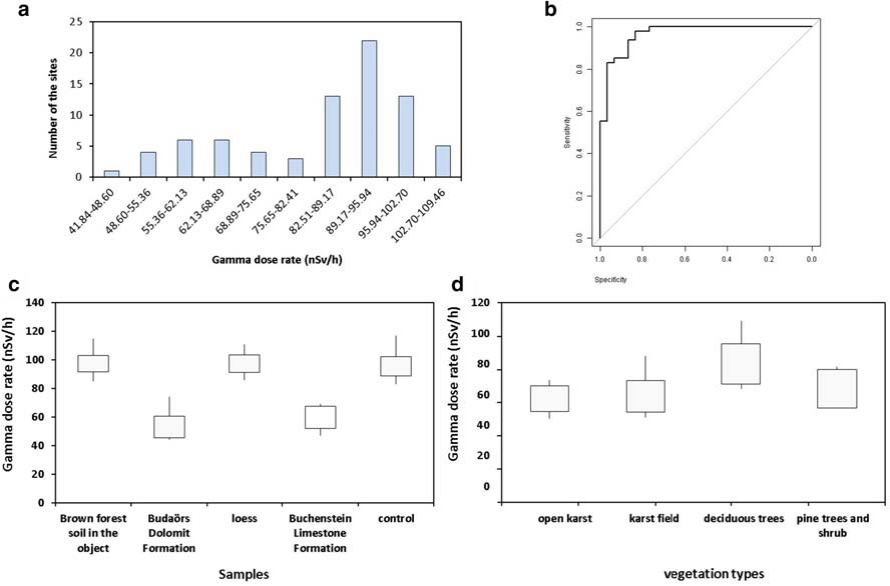
The frequency histogram of the gamma-ray dose values **a**; the ROC analysis of the field gamma-ray dose **b**; the gamma-ray dose of the different surface materials (nSv/h; **c**); the mean gamma-ray dose and the related vegetation **d**

The frequency histogram of the gamma-ray dose of the field samples shows bimodality with a peak at of 55–69 and 89–95 nSv/h gamma-ray dose. In about 27% of the sites, the mean gamma-ray dose was less than 75.6 nSv/h. In about 69% of the sites, it was more than 82.5 nSv/h (Fig. 11a). In the field survey, 47 covered and 30 opened karst surfaces were determined. According to receiver operator characteristic (ROC) analysis of the gamma dose rate of the studied karst sites, the gamma-ray dose value of 82 nSv/h is the best cut point between the covered and opened types with the AUC value of 0.966 *p* < 0.05 (Fig. 11b). The brown forest soil, the loess and the control, loess-based soil samples (mean = 99.5 nSv/h) have a 1.7 times higher mean gamma-ray dose than the mean of the Buchenstein limestone and the Budaörs dolomite (mean = 58.5 nSv/h; Fig. 11c). The mean gamma-ray dose of the open karst is 62.0 nSv/h, 69.7 nSv/h in the case of the karst fields and 71.1 above the surface of pine plantations and 88.9 in the deciduous karst forests (Fig. 11d). Based on the radiometric, the vegetation, and soil type data, a score was created to rank the environment of the paleodoline in the aspect of agriculture. The scores (from zero to three) show the increasing level of soil erosion. It can be seen that the gamma radiation data can be compared well with the characteristics of soil depth, potential vegetation, and land use possibilities (Table 3).

**Table 3:** The reconciliation of the measured gamma dose rates with the field observations of soil thickness values, the potential vegetation types, and land use

Stage of soil degradation	Soil thickness (cm)	Gamma dose rate (nSv/h)	Soil type	Potential natural vegetation	Land use
0 (preserved)	> 30	> 89	Intact brown forest soil or loess	Deciduous forest	Arable land, suitable for forestry
1 (low)	20–30	82–89	Thick soil with some carbonate debris	Xerothermic deciduous forest	Rather, suitable for forestry
2 (medium)	5–19	65–82	Thin soil with carbonate debris	Xerothermic shrub vegetation	Suitable for grazing or planting black pine forest
3 (high)	5>	65>	Bedrock with a thin skeletal soil	Open or closed rocky grassland	It has no agricultural importance

## Discussion

It is the first case when a paleodoline which is a common karst geomorphologic feature in those areas of the world—such are the Ebro Basin in Spain (Pueyo-Anchuela et al. 2010) or in the Aggtelek Karst, Hungary (Veress and Unger 2015)—where actually or in the past karstification processes took place, was investigated using the large scale of investigation tools in order to show how these objects are sensible to the anthropogenic pollution and soil degradation. The vulnerability of this karst feature can be the model of the sensitivity to the anthropogenic influence of similar karst objects.

The vertical gradients of the radionuclides and the organic material compound of the samples showed a decreasing trend according to the depth under the ground level. The probable source of the radioactive isotopes in the vertical samples is the loess sediment since each of the isotopes showed a significantly decreasing concentration trend from the surface to the floor of the paleodoline. However, it is plausible that neither the deposition of the dolomite flour nor the sedimentation of the loess was continuous and an erosion discordance between the two filling sediments can be hypothesized. Trájer et al. (2015) found that the paleodoline-filling dolomite flour can the product of erosion under the Mediterranean/warm temperate climate of the Pliocene or the early Pleistocene eras and the loess was deposited only in the late Pleistocene area, plausibly during the last glacial period.

Loessy brown forest soil and sandy loess were found to be − 2.4 m under the soil level. With a relatively sharp transition, homogenous, sandy dolomite flour, and debris were found down to the bedrock formation from − 6.7-m depth. The different isotopes showed different concentration trends. The level of the ^232^Th falls down in the loess and dolomite silt boundary, and similarly, ^40^K also suddenly sink below the 300 Bq/kg activity concentration value at the border. In contrast, ^226^Ra gradient has a continuously decreasing activity concentration trend along with the depth. These trends are related to the fact that while both the ^232^Th and the ^40^K are less and more soluble isotopes, ^226^Ra is a gas, for which this isotope can diffuse freely in the soil. The complexation of radionuclides with humic substances is also a known effect (Hummel et al. 1999). The decreasing downward trend of organic carbon primarily is related to organic material decomposition. Organic matters can migrate downward, since water-soluble humic acids nor bound by loess neither by dolomite silt. Comparing the trends of ^40^K, ^232^Th activity concentrations and the concentration of organic compounds, it can be stated that although radionuclides can be leached into the deeper layers of the soil in complexes, it may not be significant in the studied environment. It is plausible that major tectonics events and/or hydrothermal activity did not reach the structure from the start of sedimentation in the site of the borehole because the dolomite silt has low radionuclide concentrations. In fracture and fault zones, the enrichment of decay products of ^226^Ra and of ^40^K, ^238^U, and ^232^Th can be observed or due to the circulating waters (Gautam et al. 2000).

The classic field of gamma-ray dosimetry is the mapping of rocks high in thorium and uranium (Nielson et al. 1990). Although the gamma radiation intensity measurement is a useful completive tool of field geology and can be used in case of mapping several structures as subsurface karst structures like subsurface flow channels, solution cavities, and sinkholes (Gautam et al. 2000) for the mapping of underwater sediments (Povinec et al. 2008) or even the exploration of terrestrial impact craters (Vasconcelos et al. 2012) and impact paleodolines (Bose et al. 2013). Measuring of the gamma-ray dose rate of the surface soil is a useful tool in cases when the mapping of the karst and the covering or the sediment filling is requisite (Ali et al. 2011) although very few studies were performed to investigate the use of gamma radiation intensity measurements in the investigation of the covered and opened karst patterns. It has long been known fact that limestones, sandstones, and dolomites are of relatively low radioactivity (Russell 1944). In the southeastern and southwestern part of the paleodoline, the native limestone and dolomite rocks influence within a relatively short distance the gamma-ray dose. This observation indicates that in the northern part of the paleodoline, the carbonate rocks are covered by thicker sediments, mainly loess. On the side of the Buchenstein Limestone Formation, the lower radiation zone due to the debris of the limestone is wider than in the case of the dolomite rocks. It can be explained by the fact that while the rocks of the Budaörs Dolomite Formation barely rise from their relief, the rocks of Buchenstein Limestone Formation form a 20-m high hill in the southeast side of the paleodoline. Overall, the gamma-ray dose of the centrum of the paleodoline is similar to the wider areas covered by loess. Only the carbonate rocks modify the radiation landscape in the southeast and southwest parts of the paleodoline and show the signs of young denudation. It is interesting in the light of the found higher dolomite content of the soil in the southern rim of the paleodoline. This finding indicates that the loess-based matrix can increase the gamma-ray dose of the material, despite the low-level radiation of dolomite gravels. The combined measure of the carbonate content and the gamma-ray dose can help to separate the presence of the carbonate bedrock near to the surface and the carbonate debris. These results are somewhat similar to the observation of Cook et al. (1996) who found that gamma radiometry can be found as an element of airborne geophysical techniques to show the location of granite outcrops. The difference is that while granites have relatively high levels of gamma radiation from the decay of potassium, uranium, and thorium and the radiation of the covering soils are lower; in the case of partly covered karst features, the situation seems to be the inverse of the first case. In fact, covered and open karst can be well distinguished using radiometry. Our results can be used plausibly with good efficiency in other karst areas because the carbonates generally poor in radionuclides and the covering sediments are rich in heavier elements due to the selective effect of weathering.

The histogram of the gamma radiation intensity of the measured sites suggests that the bimodal curve is the composite of a two, normal distribution-like curve. This pattern can refer to the opened and the covered karst, in fact. It seems that that the gamma radiation intensity is a highly sensitive predictor of the differentiation of the opened/covered karst. The gamma radiation intensity also indirectly refers to the potential agricultural use of the area and the potential vegetation. Our results can be generalized in many areas of Europe since the low gamma radiation intensity of the bedrock can be similar in extended areas of the Alpine Triassic mountains due to the original great area of the Triassic carbonate platform of Tethys Sea. Using this principle, aerial gamma surveys could detect the pattern of the covered/opened karst patterns of larger areas. Techniques operating in a similar way are used in the determination of weathering intensity and digital terrain analysis (Wilford 2012).

The studied paleodoline has a wide but asymmetrical water catchment area (Trájer et al. 2015) which is not unusual in karstic areas since the water catchment boundaries in karst regions are often fragmented (Bonacci 2004). It was found that pollution affects mainly the south part of the paleodoline. Although the geological structure located in a non-urbanized environment, the urbanization scores showed that there are two minor urbanized areas in the narrow environment of the paleodoline. It is plausible that the source of the saline and organic carbon pollution is the adjacent farm near the southern rim of the paleodoline. The minor suburban area west from the structure does not have a direct notable pollution effect. Leaching, plowing also can be the cause of the infiltration of the southeastern parts and a potential source is an organic manure. Although minor accumulations of the organic material also can be visible in the valley of the major gullies in the east part of the slope of the paleodoline, the southern great erosion valley is the main carrier of organic materials and the pollutants into the depression. It should be noted that it is very frequent that drainage channels and gully systems associated with the paleodolines (Veress 2010). This highlights the possibility of generalizing our results for other karst areas.

The relic deciduous forest plausibly does not emit notable amounts of organic materials, which is an important result of the aspect of the protection of karst water resources. This finding can be associated with the recent climate change since the increasing summer aridity and temperature diminishes the carbonate storage of the temperate soils (Davidson and Janssens 2006; Piao et al. 2000) and the increase of the extreme meteorological events (Meehl et al. 2000) as sudden floods can allocate humic acids into the groundwater system. The recent accumulation of the probably manure-derived organic material in the central parts of the paleodoline is alarming since climate change has potential impacts on water- and foodborne diseases caused by microbiological agents (Rose et al. 2001). The irregular shape of the contamination also probably makes the effect of rain wash since minor organic carbonate enrichments were found in/close to the gullies, which proves the existence of water transport.

The lowest pH values of the soils were measured in case of soils that formed on the Buchenstein Limestone outcrop. It may indicate that the rocky substrate did not allow the agricultural use of the southeastern part of the paleodoline. This deciduous forest can be the remnant of the original vegetation probably due to the preserved original soil structure which was rich in humic acids. It is notable that the older pine plantation, which covers the eastern part of the area, still has not influenced the chemistry of the soil at − 50/55 cm under the soil level. The relative slow humidification of the pine needles and the consequent slow release of the acids and/or the naturalizing effect of the basic carbonate bedrock also can be the cause of this observation. Since the plowing is possible in the non-rocky northwest and the northeast rims of the paleodoline, it is plausible that during the decades, a great amount of basic loess was included in the depression increasing the pH in the same cardinal direction areas.

**Fig. 12:**
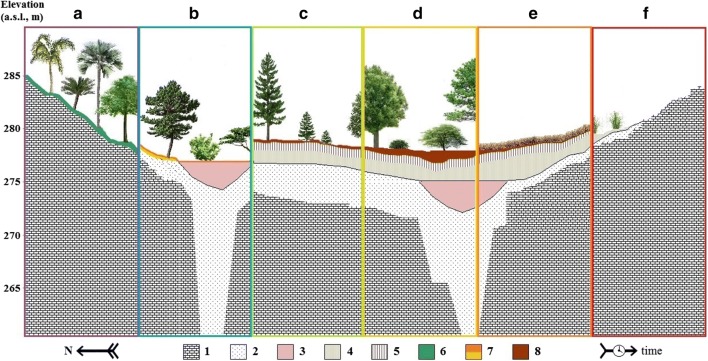
The sedimentation and development history of the Meggyespuszta paleodoline in the last 20–30 million years according to the described observations and the model of Trájer et al. (2015) (**a**: Miocene, **b**: Pliocene, **c**: late Pleistocene-early Holocene, **d**: mid-Holocene, **e**: late Holocene, **f**: Anthropocene; 1: carbonate bedrock, 2: dolomite silt, 3: red bauxitic clay (fossilized laterite), 4: loess, 5: podsol or brown forest soil, **a** level, 6: skeletal soil, 7: laterite soil, 8: podsol or brown forest soil, **a** level)

The studied paleodoline originally could be a covered karst structure during the Holocene. The large karst feature itself plausibly was formed under the humid and wet climatic conditions of the Miocene epoch (Fig. 12a). In the Pliocene, the climate became drier and the dolomite silt was leached into the depression. The remains of the laterite soil preserved in the deepest parts of the paleodoline according to the results of the former drilling projects (Fig. 12b). The exact time of the sedimentation of the loess cover is not known, but the lack of the well-detectable paleosoil horizons suggests that it was deposited only in the Last Glacial Period (Trájer et al. 2015; Fig. 12c). In the last two thousand years, humans modified the karst formations by deforestation, agricultural land use, and mining of the Balaton Highland. The deforestation of the karst plateau occurred in the Middle Age and the affected area lost its loess and soil cover only in the dawn of the historical New Age. Till the eighteenth century, the major part of the Meggyespuszta paleodoline was covered by oak forest with mainly Turkish oak and Downy oak (Fig. 12d). By the end of the Middle Ages, the north and northeastern part of the Veszprém Plain, the wider environment of the Meggyespuszta paleodoline and the southern rim of the paleodoline became partly a rocky, treeless, or shrubby land losing its loess and silt cover (Fig. 12e, f). Similar changes transformed the originally widely forested Mediterranean areas into the recently known dry, degraded landscape under the late Roman times and the Middle Ages (Gams 1993). In the nineteenth to twentieth century, the re-establishment of the forests was attempted by plantation of *Pinus nigra* between Veszprém and Várpalota and had highly variable partial success.

## Conclusions

The reconstructed sedimentation and development history of the paleodoline indicates that after the Miocene epoch, episodical sedimentation processes characterized the geological evolution of the studied karstic depression until the appearance of human influence. Field gamma-ray dose measurement can explore the spatial patterns of the covered and uncovered karsts. Because in the studied geological environment, the loess sediment is the parent material of the radionuclides of the soil, the method is not able to detect the early phases of soil degradation. The field gamma-ray dose patterns are in accordance with the potential land use and vegetation of a site. Our results indicate that such paleodolines like the Meggyespuszta paleodoline should be taken out of agricultural cultivation to prevent the karst aquifer from the contaminants. It can be concluded that paleodolines are highly vulnerable to anthropogenic impacts and karst aquifers can only be preserved with the strict protection of these structures. The most effective way to prevent soil erosion would be to reforest the edges and the catchment area. The establishment of a buffer zone would also prevent from entering of agricultural contaminants the karst catchment.
